# Long-lasting reduction of risk of colorectal cancer following screening endoscopy

**DOI:** 10.1054/bjoc.2001.2023

**Published:** 2001-09

**Authors:** H Brenner, V Arndt, T Stürmer, C Stegmaier, H Ziegler, G Dhom

**Affiliations:** Department of Epidemiology, German Centre for Research on Ageing, Bergheimer Str. 20, Heidelberg, D-69115, Germany; Department of Epidemiology, University of Ulm, Ulm, D-89069, Germany; Saarland Cancer Registry, Virchowstr. 7, Saarbrücken, D-66119, Germany; Am Webersberg 20, Homburg/Saar, D-66424, Germany

**Keywords:** colonoscopy, colorectal cancer, endoscopy, risk, sigmoidoscopy

## Abstract

Several studies have suggested that incidence and mortality of colorectal cancer (CRC) may be strongly reduced for up to 10 years by endoscopic screening with removal of precancerous lesions, but so far there are no data on risk reduction beyond this period. We assessed long-term reduction of CRC risk following screening endoscopy in a statewide population-based case–control study in Saarland, Germany. Lifetime history of screening endoscopy was compared between 320 cases with CRC aged 45–80 and 263 controls with other forms of cancer recruited from the same population. Potential confounding factors were controlled for by multiple logistic regression. 11% of cases compared to 27% of controls had a history of endoscopy for screening purposes (adjusted odds ratio (OR) = 0.28, 95% confidence interval (CI): 0.16–0.48). This strong risk reduction was also seen (OR = 0.41, 95% CI: 0.19–0.89) in subjects who had their last screening endoscopy more than 10 years ago (median: 18.9 years). Long term (> 10 years since last screening) risk reduction appeared to be particularly strong for advanced (Dukes C or D) CRC (OR = 0.19, 95% CI: 0.06–0.64). We conclude that risk reduction by screening endoscopy is long lasting, in particular with respect to advanced CRC. © 2001 Cancer Research Campaignhttp://www.bjcancer.com


British Journal of Cancer (2001) 85(7), 972–976
© 2001 Cancer Research Campaign
doi: 10.1054/ bjoc.2001.2023, available online at http://www.idealibrary.com on IDEAL

http://www.bjcancer.com

### Long-lasting reduction of risk of colorectal cancer following screening endoscopy

**H Brenner[1,2], V Arndt[2], T Stürmer[1,2], C Stegmaier[3], H Ziegler[3] and G Dhom[4]**

[1]Department of Epidemiology, German Centre for Research on Ageing, Bergheimer Str. 20, D-69115 Heidelberg, Germany; [2]Department of Epidemiology, University of Ulm, D-89069 Ulm, Germany; [3]Saarland Cancer Registry, Virchowstr. 7, D-66119 Saarbrücken, Germany; [4]Am Webersberg 20, D-66424 Homburg/Saar, Germany

**Summary** Several studies have suggested that incidence and mortality of colorectal cancer (CRC) may be strongly reduced for up to 10 years by endoscopic screening with removal of precancerous lesions, but so far there are no data on risk reduction beyond this period. We assessed long-term reduction of CRC risk following screening endoscopy in a statewide population-based case–control study in Saarland, Germany. Lifetime history of screening endoscopy was compared between 320 cases with CRC aged 45–80 and 263 controls with other forms of cancer recruited from the same population. Potential confounding factors were controlled for by multiple logistic regression. 11% of cases compared to 27% of controls had a history of endoscopy for screening purposes (adjusted odds ratio (OR) = 0.28, 95% confidence interval (CI): 0.16–0.48). This strong risk reduction was also seen (OR = 0.41, 95% CI: 0.19–0.89) in subjects who had their last screening endoscopy more than 10 years ago (median: 18.9 years). Long term (> 10 years since last screening) risk reduction appeared to be particularly strong for advanced (Dukes C or D) CRC (OR = 0.19, 95% CI: 0.06–0.64). We conclude that risk reduction by screening endoscopy is long lasting, in particular with respect to advanced CRC. © 2001 Cancer Research Campaign http://www.bjcancer.com

**Keywords**: colonoscopy; colorectal cancer; endoscopy; risk; sigmoidoscopy

Colorectal cancer (CRC) is the third commonest cancer in the world, accounting for about 9% of new cancer cases per year (Schottenfeld and Winawer, 1996). The putative very long duration of the adenoma to carcinoma sequence gives an ample opportunity to detect and treat precursors of CRC, and to reduce incidence and mortality from CRC (Gore, 1997).

Several screening methods are currently available and recommended to a varying degree in different countries (Anwar et al, 1998; Bond, 1999; Read and Kodner, 1999). They include faecal occult blood testing (FOBT), digital rectal examination, double-contrast barium enema, flexible or rigid sigmoidoscopy and colonoscopy. So far, results from large-scale prospective randomized trials are available only for FOBT, with reduction of CRC mortality by up to one third for annual FOBT screening (Towler et al, 1998; Mandel et al, 1999). The potential for reduction of CRC incidence and mortality is probably considerably higher for the endoscopic examinations with removal of early cancers or precancerous lesions as needed. Several epidemiologic studies from the US (Gilbertsen and Nelms, 1978; Newcomb et al, 1992; Selby et al, 1992; Winawer et al, 1993; Müller and Sonnenberg, 1995; Slattery et al, 2000) and one small randomized trial from Norway (Thiis-Evensen et al, 1999) have shown substantial risk reduction for up to 10 years following sigmoidoscopy or colonoscopy, but so far there are no data on risk reduction beyond 10 years after endoscopy. Large-scale randomized trials have recently been initiated to assess prevention of CRC morbidity and mortality by screening endoscopy, but results on long-term effects will not be available for another 10 or 20 years.

*Received 20 March 2001*
*Revised 30 May 2001*
*Accepted 19 June 2001*

*Correspondence to:* H Brenner

The aim of this case–control study from Germany was to assess reduction of CRC risk over a long-term period (up to and beyond 10 years) following a single screening endoscopy.

### MATERIALS AND METHODS

### Study design and study population

A population-based, statewide study on the use of screening and diagnostic procedures was conducted among patients with various forms of cancer in Saarland, a state with about one million inhabitants in Western Germany (**Ver**lauf der **di**agnostischen Abklärung, VERDI). Details of the study, which was primarily designed to address factors determining diagnostic delay and late stage diagnosis, have been reported elsewhere (Arndt et al, 2001). Briefly, residents of Saarland with a first diagnosis of colorectal cancer (CRC), gastric cancer and breast cancer below age 80 between November 1996 and February 1998 were recruited. 34 of 36 hospitals in Saarland and the adjacent counties participated in recruitment. Among 957 eligible patients recruited by these hospitals, 16 patients (1.7%) could not be enrolled because they died before an interview could be scheduled, and another 33 patients (3.4%) because they did not agree to participate. Overall, 908 patients (439 patients with CRC, 82 patients with gastric cancer and 387 patients with breast cancer) living in Saarland were enrolled. The study was approved by the Ethics Committees of the University of Ulm and of the Physicians' Boards of the state of Saarland and the adjacent state of Rheinland Pfalz.

In this case–control analysis previous history of screening endoscopy was compared between patients with CRC and patients with the other forms of cancer. Separate and joint analyses were carried out for women and men. For both sexes, the case group included patients aged 45–80 with a first diagnosis of colorectal cancer (ICD-9 153, 154). Patients below 45 years were excluded because CRC is very rare and screening measures are typically only considered among special risk groups in this age group. For women, the control group consisted of those with breast cancer (ICD-9 174) within the same age range. For men, the control group consisted of 45–80-year-old men with gastric cancer (ICD-9 151).

To avoid potential detection bias resulting from increased detection of tumours among subjects more ready to participate in screening examinations and because the endpoint of interest was reduction of clinically manifest CRC, we further excluded 175 patients (79 cases, 96 controls) whose cancer was detected by screening measures or incidentally (e.g. in the context of another health examination). We excluded 6 subjects with a history of inflammatory bowel disease and another 11 patients with a history of more than one screening endoscopy within the previous 5 years, because these patients might have undergone repeat endoscopic screening examinations due to increased risk of CRC. Screening history was missing for 19 patients, leading to a final sample size of 320 cases and 263 controls for this analysis.

### Data collection

Patients were informed about the study by their physicians during the first hospitalization with cancer, typically several days to weeks after initial treatment. After informed consent was obtained, patients were reported to the study center in Saarbrücken, the capital of Saarland, and interviews were scheduled to be conducted by one of three trained interviewers during hospitalization.

Interviews were conducted following a standardized questionnaire. Questions included detailed information on medical history, symptoms, diagnostic measures, previous screening examinations, sociodemographic factors, and potential risk factors of the cancer. Detailed information on the onset of the first symptoms leading to the current cancer diagnosis was obtained. Patients were asked if they had had a large bowel endoscopy for screening purposes in the past, that is, before the onset of the first symptoms leading to the current cancer diagnosis. No distinction was made between primary screening by endoscopy, or endoscopic screening following another screening examination, e.g. a positive FOBT. In case of a history of endoscopic screening, the time of the last screening endoscopy was obtained, and the time between the last screening endoscopy and the current cancer diagnosis was calculated. No distinction between flexible or rigid sigmoidoscopy and colonoscopy was made.

Only examinations that were unrelated to the recent cancer diagnosis, i.e. endoscopies that were done before the onset of the first symptoms leading to the current cancer diagnosis and those explicitly conducted for screening purposes were considered for this analysis. Therefore, and because only cases with a first diagnosis of CRC were included, our results show risk reduction following a negative screening endoscopy (that is, endoscopy without identification of CRC, but with potential identification and removal of precancerous lesions). Information on cancer location, spread and histology was extracted from the medical records.

### Statistical methods

We first described cases and controls with respect to basic clinical and sociodemographic characteristics and potential risk factors for CRC.

We then compared the history of screening endoscopy between cases and controls. In all analyses, subjects without a history of screening endoscopy formed the reference group. The association between history of endoscopy and risk of CRC was quantified by time since last screening endoscopy (≤ 5 years/5–10 years/≥10 years) using odds ratios and their 95% confidence intervals (Weiss et al, 1992). Multiple logistic regression was employed to adjust associations for potential confounding factors. The following covariates which were known or suspected to be related to the risk of CRC and which might also be related to history of screening endoscopy were considered: age (in years), family history (history of CRC in a first degree relative), school education (≤ 9 years/> 9 years), marital status (married/other), smoking (current/former/never) and alcohol consumption (no/yes). To take general health behaviour into account, we further controlled for history of a general health examination, a screening examination focusing on cardiovascular disease, diabetes and nephropathy offered every two years to adults above age 36 in Germany since 1989.

In addition to the main analyses, separate analyses were carried out for early (Dukes A or B) and advanced (Dukes C or D) CRC and for rectosigmoidal and other CRC.

**Table 1** Characteristics of cases and controls

| | Women | | Men | | Total | |
|---|---|---|---|---|---|---|
| | Cases | Controls | Cases | Controls | Cases | Controls |
| Tumour site | colon/rectum | breast | colon/rectum | stomach | colon/rectum | breast/stomach |
| Number of patients | *n* = 132 | *n* = 229 | *n* = 188 | *n* = 34 | *n* = 320 | *n* = 263 |
| Age (mean) | 65.7 | 61.3 | 65.4 | 65.2 | 65.5 | 61.8 |
| Education > 9 years | 11% | 18% | 24% | 9% | 19% | 16% |
| Married | 51% | 58% | 87% | 82% | 72% | 61% |
| Family history of CRC | 14% | 5% | 15% | 6% | 15% | 5% |
| Smoking former | 23% | 22% | 62% | 64% | 46% | 28% |
| Current | 11% | 11% | 14% | 18% | 13% | 12% |
| Alcohol consumption | 85% | 86% | 94% | 88% | 90% | 86% |
| Participation in general health examination | 62% | 62% | 68% | 68% | 65% | 63% |

*© 2001 Cancer Research Campaign* 

### RESULTS

Overall, 320 patients with CRC aged 45–80 were included in this analysis (132 women, 188 men, Table 1). The proportions of CRC patients with localized cancer (Dukes A and B), regional (Dukes C) and distant (Dukes D) tumour spread were 54%, 28% and 19%, respectively. Controls were 229 women with breast cancer and 34 men with stomach cancer. The median time between onset of symptoms and definite diagnosis was 2 months for CRC cases, 1 month for breast cancer controls and 3 months for gastric cancer controls. Mean age of CRC cases was about 66 years (Table 1). Both female and male controls were on average somewhat younger (61 and 65 years, respectively). The proportion of married subjects and of former smokers were higher among men than among women, but there were only minor differences between cases and controls within both genders (the difference in the proportion of married women is essentially due to the age difference between cases and controls). Current smoking was rare, and alcohol consumption was common among cases and controls in both women and men. About two third of all participants reported at least one previous general health examination, with very little differences between cases and controls. By contrast, a major difference between cases and controls was found for a family history of CRC (15% in cases, 5% in controls), which reflects the well known familial aggregation of CRC.

A previous history of screening endoscopy was more often reported by controls (27%) than by cases (11%) (Table 2). This pattern was observed in both genders, and further analyses were combined for both genders. A pooled estimate of the odds ratio (OR) of 0.30 (95% confidence interval, CI: 0.18–0.50) was obtained for the association between history of a negative screening endoscopy and risk of CRC after adjustment for gender. This estimate was essentially unchanged by additional control for the other covariates in multiple logistic regression (OR = 0.28, 95% CI: 0.16–0.48).

In Table 3, analyses are further stratified by time since last screening endoscopy. Although risk reduction was strongest (about 80%) for subjects with a screening endoscopy within the last 10 years (with little difference between time windows ≦ 5 and 5–10 years), a considerable risk reduction by about 60% was also seen for subjects who had their last endoscopy more than 10 years ago (median 18.9 years).

Further analyses by tumour spread suggested long-term (> 10 years) risk reduction to be particularly strong for advanced tumour stages Dukes C and D (OR = 0.19; 95% CI: 0.06–0.64), and both short- (≦ 10 years) and long-term (> 10 years) risk reduction to be stronger for rectosigmoid (OR = 0.21; 95% CI: 0.09–0.46 and OR = 0.34; 95% CI: 0.14–0.87, respectively) than for other large bowel cancers (OR = 0.29; 95% CI: 0.11–0.77 and OR = 0.70; 95% CI: 0.26–1.86). However, these results among subgroups of cases need to be interpreted with caution given the width of the confidence intervals.

### DISCUSSION

Our results are consistent with and extend findings of several epidemiologic studies from the United States which reported a strong reduction of incidence and mortality of distal CRC within up to 10 years following screening endoscopy (Gilbertsen and Nelms, 1978; Newcomb et al, 1992; Selby et al, 1992; Müller and Sonnenberg, 1995; Slattery et al, 2000). To our knowledge, this is the first study that addressed risk reduction beyond 10 years following screening endoscopy, and it revealed that a strong reduction of CRC risk by about 60% prevails well beyond 10 years indeed.

Our study included virtually all hospitals and the majority of patients within a defined geographic area. This approach implies that the results probably more adequately reflect possible risk reduction on the population level than the often somewhat more

**Table 2** History of screening endoscopy among cases and controls

| | Women | | Men | | Total | |
|---|---|---|---|---|---|---|
| | **Cases** *n* = 132 | **Controls** *n* = 229 | **Cases** *n* = 188 | **Controls** *n* = 34 | **Cases** *n* = 320 | **Controls** *n* = 263 |
| History of screening endoscopy | 10 (8%) | 62 (27%) | 25 (13%) | 8 (24%) | 35 (11%) | 70 (27%) |
| OR (95% CI)[a] | 0.22 (0.11–0.45) | | 0.50 (0.20–1.22) | | 0.30 (0.18–0.50)[b]<br>0.28 (0.16–0.48)[c] | |

[a]Odds ratio (95% confidence interval); [b]Adjusted for gender; [c]additionally adjusted for age, family history of colorectal cancer, school education, marital status, smoking, alcohol consumption, and participation in general health examination.

**Table 3** Time since last screening endoscopy among cases and controls

| Time since last screening endoscopy | | | | Odds ratio (95% confidence interval) | |
|---|---|---|---|---|---|
| **Interval** | **Median** | **Cases** *n* = 320 | **Controls** *n* = 263 | **Adjusted for gender** | **Adjusted for multiple covariates**[a] |
| No endoscopy | – | 89.1% | 73.4% | 1.0[reference] | 1.0[reference] |
| ≤ 5 y | 2.9 y | 3.4% | 10.3% | 0.27 (0.12–0.62) | 0.20 (0.08–0.49) |
| 5–10 y | 7.0 y | 2.5% | 7.2% | 0.23 (0.09–0.60) | 0.23 (0.08–0.66) |
| > 10 y | 18.9 y | 5.0% | 9.1% | 0.40 (0.19–0.85) | 0.41 (0.19–0.89) |

[a]Adjusted for gender, age, family history of colorectal cancer, school education, marital status, smoking, alcohol consumption, and participation in general health examination.

 *© 2001 Cancer Research Campaign*

optimistic results obtained from highly specialized clinical settings. Because subjects who were too sick to be interviewed were not eligible for recruitment (and another – though very small – proportion of patients died before interviews could be scheduled), subjects with advanced cancers are likely to be somewhat under-represented in our sample. As long-term risk reduction appears to be even stronger for advanced than for early cancers, this may have led to some underestimation of long-term risk reduction following a screening endoscopy.

We used cancer rather than population controls. The validity of the results therefore depends on the assumption that patients with breast and stomach cancer do not differ from the general population with respect to history of screening endoscopy (but not on the assumption that these patients do not differ from the general population with respect to CRC risk). There is no obvious reason, why a history of previous large bowel screening endoscopy (performed before onset of first symptoms) should be related to breast cancer or stomach cancer. This point is supported by the consistency of our findings with other, population-based studies that have assessed risk reduction within 10 years following screening endoscopy. The fact that participation in general health screening examinations was virtually identical in cases and controls in our study suggests that bias due to differential health consciousness is also unlikely.

An advantage to the use of cancer rather than population controls is that interviews could be realized in exactly the same setting by the same trained interviewers following a standardized questionnaire which should reduce bias due to differential recall, a major problem in case–control studies relying on population controls.

History of screening endoscopy in this German population does reflect individual patient management rather than screening within the context of a primary endoscopic screening programme. A nationwide screening programme for CRC offering annual FOBT and digital rectal exam to women and men above 45 years was introduced in Germany in 1977 (Wahrendorf et al, 1993). The programme does not include endoscopy as primary screening tool, but positive FOBT results are typically followed up by colonoscopy. History of screening endoscopy reported in this study includes, but is not restricted to evaluation of positive FOBT screening results. Because patients with increased risk of CRC may be more prone to undergo screening endoscopy, we excluded patients with a history of inflammatory bowel disease, and carefully controlled for indicators of increased risk of CRC, such as a positive family history. We cannot exclude, however, that the risk reduction following screening endoscopy might have been underestimated to some extent due to residual confounding.

Our results show risk reduction following a 'negative' screening endoscopy, that is, endoscopy without identification of CRC, but with potential identification and removal of precancerous lesions. This has to be distinguished from the potential additional benefit of 'positive' screening endoscopy, which would be mediated by early treatment of screening detected invasive CRC (Weiss, 1999; Imperiale et al, 2000; Lieberman et al, 2000). The definitive goal of cancer screening is to reduce cancer mortality, and therefore fatal cancer is the most important endpoint for evaluation of potential screening examinations. For CRC, mortality is strongly related to stage at diagnosis, with 5-year relative survival rates ranging from 80–90% for Dukes A and B tumours to about 5% for Dukes D tumours (Wingo et al, 1998). In our analysis, overall risk reduction was almost identical for Dukes A and B and Dukes C and D tumours, and long-term risk reduction appeared to be particularly strong for the latter. This suggests that long-term reduction of fatal CRC following negative screening endoscopy should be at least as strong as reduction of incident CRC.

A limitation of our study is that history of screening endoscopy was based on self-reports and could not be validated by medical records, a difficulty commonly encountered in epidemiologic studies looking at lifetime history of medical procedures (a substantial proportion of which dates back a very long time). However, screening history was carefully ascertained by trained medical staff in our study. Furthermore, large bowel endoscopy is an invasive procedure that should be well remembered by most patients, and high validity of self reported colorectal cancer screening history has been demonstrated empirically (Gordon et al, 1993; Baier et al, 2000). However, no reliable information about findings at screening examinations could be obtained, and no definite distinction could be made between sigmoidoscopy and colonoscopy. The pattern of a less strong risk reduction for proximal colon cancer than for rectosigmoid cancer appears plausible given that no risk reduction for proximal colon cancer would be expected among those study participants who underwent sigmoidoscopy only. This implies, on the other hand, that reduction of proximal colon cancer might be much stronger in the subgroup of patients who underwent colonoscopy.

This is the first study on long-term effects of screening endoscopy, and its results have to be replicated in other populations. If confirmed in other studies, our results may have important public health implications: firstly, our results support the case for implementation and evaluation of endoscopy as primary screening tools in CRC screening programmes (Atkin, 1998; Lieberman, 1998). Unlike the FOBT, which often fails to identify patients with precancerous lesions, large bowel endoscopy has the potential of substantial CRC prevention through identification and removal of adenomatous polyps. Secondly, our analysis supports the suggestion, that the strong protective effect of endoscopic screening with removal of precancerous lesions is long-lasting, and that this effect may be achieved with long-term screening intervals. In particular, our results confirm and extend the notion that a follow-up endoscopy would usually not be necessary for at least another 10 years unless there are specific indications for it (Atkin et al, 1992; Hoff et al, 1996). Very effective prevention of advanced CRC manifestations which account for the vast majority of CRC deaths may even be achieved with longer screening intervals. The possibility to extend screening intervals to 10 years or even longer suggested by our results might strongly enhance the feasibility, acceptance, reduction of side effects and cost effectiveness of population-wide endoscopic screening (Sonnenberg et al, 2000). This particularly applies to colonoscopy which is more invasive than other screening procedures, but which provides the possibility to detect and remove precancerous lesions in the entire large bowel.

### ACKNOWLEDGEMENTS

The authors are highly indebted to the willingness of patients to participate in this study and to Annelie Becker, Corinna Hetke, Wiebke Michaels and Marianne Schramm for conducting the patient interviews. We would also like to thank Rainer Müller, Rolf Friemond, Dietlind Wehrhahn and Daniela Österle for their excellent technical assistance. This study was partly funded by the German Cancer Foundation (Deutsche Krebshilfe; Project Number 70-1816).

*© 2001 Cancer Research Campaign*

### REFERENCES

Anwar S, Hall C and Elder JB (1998) Screening for colorectal cancer: present, past and future. *Eur J Surg Oncol* **24**: 477–486

Arndt V, Stürmer T, Stegmaier C, Ziegler H, Dhom G and Brenner H (2001) Sociodemographic factors, health behavior and late stage diagnosis of breast cancer in Germany – a population based study. *J Clin Epidemiol* **54**: 719–727

Atkin WS (1998) Flexible sigmoidoscopy as a mass screening tool. *Eur J Gastroenterol Hepatol* **10**: 219–223

Atkin WS, Morson BC and Cuzick J (1992) Long-term risk of colorectal cancer after excision of rectosigmoid adenomas. *N Engl J Med* **326**: 658–662

Baier M, Calonge N, Cutter G, McClatchey M, Schoentgen S, Hines S, Marcus A and Ahnen D (2000) Validity of self-reported colorectal cancer screening behavior. *Cancer Epidemiol Biomarkers Prev* **9**: 229–232

Bond JH (1999) Screening guidelines for colorectal cancer. *Am J Med* **106**(1A): 7S–10S

Gilbertsen VA and Nelms JM (1978) The prevention of invasive cancer of the rectum. *Cancer* **41**: 1137–1139

Gordon N, Hiatt R and Lampert D (1993) Concordance of self-reported data and medical record audit for six cancer screening procedures. *J Natl Cancer Inst* **85**: 566–570

Gore RM (1997) Colorectal cancer. Clinical and pathological features. *Radiol Clin North Am* **35**: 403–429

Hoff G, Sauar J, Vatn MH, Larsen S, Langmark F, Moen IE, Foerster A and Thiis-Evensen E (1996) Polypectomy in prevention of colorectal cancer: 10 years follow-up of the Telemark Polyp Study I (TPS-I). A prospective, controlled population study. *Scand J Gastroenterol* **31**: 1006–1010

Imperiale TF, Wagner DR, Lin CY, Larkin GN, Rogge JD and Ransohoff DF (2000) Risk of advanced proximal neoplasms in asymptomatic adults according to the distal colorectal findings. *N Engl J Med* **343**: 169–174

Lieberman D (1998) Colonoscopy as a mass screening tool. *Eur J Gastroenterol Hepatol* **10**: 225–229

Lieberman DA, Weiss DG, Bond JH, Ahnen DJ, Garewal H and Chejfec G (2000) Use of colonoscopy to screen asymptomatic adults for colorectal cancer. *N Engl J Med* **343**: 162–168

Mandel JS, Church TR, Ederer F and Bond JH (1999) Colorectal cancer mortality: effectiveness of biennial screening for fecal occult blood. *J Natl Cancer Inst* **91**: 434–437

Müller AD and Sonnenberg A (1995) Protection by endoscopy against death from colorectal cancer. A case control study among veterans. *Arch Intern Med* **155**: 1741–1748

Newcomb PA, Norfleet RG, Storer BE, Surawicz TS and Marcus PM (1992) Screening sigmoidoscopy and colorectal cancer mortality. *J Natl Cancer Inst* **84**: 1572–1575

Read TE and Kodner IJ (1999) Colorectal cancer: risk factors and recommendations for early detection. *Am Fam Physician* **59**: 3083–3092

Schottenfeld D and Winawer SJ (1996) Cancers of the large intestine. In Cancer Epidemiology and Prevention, 2nd ed, Schottenfeld D, Fraumeni JF Jr (eds) pp 813–840. Oxford University Press: New York, Oxford

Selby JV, Friedman GD, Quesenberry CP and Weiss NS (1992) A case-control study of screening sigmoidoscopy and mortality from colorectal cancer. *N Engl J Med* **326**: 653–657

Slattery ML, Edwards SL, Ma KN and Friedman GD (2000) Colon cancer screening, lifestyle, and risk of colon cancer. *Cancer Causes & Control* **11**: 555–563

Sonnenberg A, Delcòf and Inadomi JM (2000) Cost-effectiveness of colonoscopy in screening for colorectal cancer. *Ann Intern Med* **133**: 573–584

Thiis-Evensen E, Hoff GS, Sauar J, Langmark F, Majak BM and Vatn MH (1999) Population-based surveillance by colonoscopy: effect on the incidence of colorectal cancer. Telemark Polyp Study I. *Scand J Gastroenterol* **34**: 414–420

Towler B, Irwig L, Glasziou P, Kewenter J, Weller D and Silagy C (1998) A systematic review of the effects of screening for colorectal cancer using the faecal occult blood test, Hemoccult. *Br Med J* **317**: 559–565

Wahrendorf J, Robra BP, Wiebelt H, Oberhausen R, Weiland M and Dhom G (1993) Effectiveness of colorectal cancer screening: results from a population-based evaluation in Saarland, Germany. *Eur J Cancer Prev* **2**: 221–227

Weiss NS (1994) Application of the case-control method in the evaluation of screening. *Epidemiol Rev* **16**: 102–108

Weiss NS (1999) Case-control studies of the efficacy of screening tests designed to prevent the incidence of cancer. *Am J Epidemiol* **149**: 1–4

Weiss NS, McKnight B and Stevens NG (1992) Approaches to the analysis of case-control studies of the efficacy of screening for cancer. *Am J Epidemiol* **135**: 817–823

Winawer SJ, Zauber AG, Ho MN, O'Brien MJ, Gottlieb LS, Sternberg SS et al (1993) Prevention of colorectal cancer by colonoscopic polypectomy. *N Engl J Med* **329**: 1977–1981

Wingo PA, Gloeckler Ries LA, Parker SL and Heath CW (1998) Long-term cancer patient survival in the United States. *Cancer Epidemiol Biomarkers Prev* **7**: 271–282

*© 2001 Cancer Research Campaign*
